# Effects of exposure to body-positive advertising on body image among young Chinese women

**DOI:** 10.3389/fpsyg.2026.1731086

**Published:** 2026-02-04

**Authors:** Jingjing Nie

**Affiliations:** School of Journalism and Communication, Wuhan University, Wuhan, China

**Keywords:** body image, body-positivity, Chinese female, commercialization, social media

## Abstract

**Introduction:**

The commercialization of the Body Positivity (BoPo) movement on social media platforms has sparked ongoing debate regarding its impact on audience body image.

**Methods:**

This study employs a randomized controlled online experiment, recruiting 172 young Chinese women (aged 18–30) and randomly allocating them into three groups: a body-positive advertising group, ideal beauty (thin-ideal) advertising group, and an appearance-neutral post group featuring natural scenery.

**Results:**

The results indicate that exposure to brand-posted BoPo advertisements significantly increased participants’ positive mood, body satisfaction, and body appreciation compared to both the ideal beauty advertisement and the appearance-neutral post groups, further supporting core tenets of Positive Body Image theory. Notably, BoPo advertisements also resulted in a reduction in self-objectification levels. This finding contrasts with some existing literature and suggests that body-positive content may operate through a more complex and potentially more positive psychological mechanism within the context of social media commercialization.

**Discussion:**

Overall, this research demonstrates a significant positive influence of commercialized BoPo content on the body image of young women. It provides important theoretical and empirical evidence for further understanding and evaluating the evolving commercialization of the Body Positivity movement and contributes to the understanding of its psychological mechanisms in a commercial context.

## 1 Introduction

In response to the pervasive realities of body image concerns and body cognitive dissonance among young people (Jackson et al., 2020; Zhang et al., 2018), a movement called “body positivity” has emerged and spread globally. Existing research has demonstrated that body-positive content on social media platforms has a significant positive impact on women (Cohen et al., 2019a; Rousseau, 2023; Stevens and Griffiths, 2020). Influenced by this movement, a noteworthy shift has emerged in the Chinese social media ecosystem: a growing number of brands are advocating for BoPo, aiming to communicate positive body image messages and alleviate the widespread body anxiety experienced by young Chinese female consumers. However, the commercialization of body positivity has created new contradictions. Critics argue that empowerment marketing, leveraging social movements, has not transcended its consumerist essence. While it appears to promote social equality and transformation, such marketing strategies often remain superficial and have limited substantive impact (Brathwaite and DeAndrea, 2022; Cwynar-Horta, 2016). However, current research has not conducted an in-depth empirical analysis of body-positive marketing on social media from the brand perspective. Furthermore, within the Chinese context, the dissemination of remains in its early stages. Compared with young women in Western societies, Chinese young women’s body image is shaped by the interplay of cultural traditions, social expectations, and modern media influences, resulting in a general tendency toward body dissatisfaction and negative body image (Lang and Ye, 2024). Therefore, this study deeply investigates the core question: Can BoPo advertisements posted by brands on social media genuinely enhance the body image of young Chinese women?

## 2 Literature review

### 2.1 The body positivity movement and Chinese social media

“Ideal beauty” has long been accepted and embraced by the public as mainstream (Cohen et al., 2019a). Influenced by mass media, the “ideal beauty” commonly embraced by young Chinese women typically manifests as a set of physical characteristics represented by “Pale, Young, and Slim” (Liu and Li, 2024): double eyelids and large eyes, a high and straight nose bridge, a melon-seed-shaped face, a well-defined jawline, and fair skin, complemented by a slender (approximately 20 pounds below average weight) and tall (approximately 3 to 5 inches above average height) figure. Among these features, fair skin, a melon-seed-shaped face, and a slender physique reflect traditional Asian aesthetic preferences. In contrast, large eyes, a high nose bridge, and a tall stature, to some extent, reflect the influence of Western or modern aesthetic concepts (Zhang, 2012). However, studies have shown that media representations of “ideal beauty” have negative effects on young women’s body image. According to sociocultural theory, the public internalizes unrealistic “ideal beauty” standards conveyed by the media and engages in upward social comparison (Thompson et al., 1999), leading to body dissatisfaction (Cohen et al., 2019a), body disorder (Grabe et al., 2008), and psychological disorders such as depression and eating disorders (O’Brien et al., 2009). At the same time, the socio-cultural significance symbolized by the “ideal beauty” also influences youth groups. For instance, obesity is often perceived as unattractive, lacking in appeal, and unhealthy (Team V, 2020).

To challenge the dominant social norms of “ideal beauty” constructed by the media, the body positivity movement emerged on Instagram in 2012 and gained popularity among young people (Cwynar-Horta, 2016). The body positivity movement aims to challenge the current “thin-ideal” and “fit-ideal” body ideals, celebrating the beauty of diverse shapes, sizes, colors, features, and abilities, and emphasizing self-appreciation and self-acceptance (Cohen et al., 2019a; Cohen et al., 2019b; Cwynar-Horta, 2016). This movement originated from the feminist-based fat acceptance movement of the 1960s, which arose in response to the growing anti-fat discourse prevalent in Canada and the United States during that period (Afful and Ricciardelli, 2015; Cohen et al., 2019b). The emergence of the body positivity movement has disrupted the single, socially dominant appearance standard and increased the visibility of marginalized groups (such as people with disabilities and those with obesity).

Body positivity on social media has a positive impact. Research demonstrates that exposure to body-positive content on social media increases body satisfaction (Cohen et al., 2019a; Nelson et al., 2022; Simon and Hurst, 2021) and fosters higher positive mood. Not only are these short-term effects significant, but exposure to body-positive content on social media also has positive long-term effects (Stevens and Griffiths, 2020). A 28-day ecological momentary experiment among young Italian women found that daily exposure to body-positive Instagram content significantly improved mood and body image compared to exposure to “ideal beauty” content (Fioravanti et al., 2023). Similar findings were also found among young Australians and Asians (Rodgers et al., 2022). However, as the Australian sample included Asian women, that study did not directly compare young people from different regions. Another study comparing Chinese and Croatian youth found that Chinese women reported higher levels of body dissatisfaction, greater social pressure, and stronger internalization of the “ideal beauty” standard (Stojcic et al., 2020). Findings indicate that cultural contexts significantly influence preferences for ideal body types and body satisfaction (Stojcic et al., 2020). Compared to European countries, Asian nations exhibit higher rates of body image dissatisfaction (Holmqvist and Frisén, 2010). This aligns with the reality that young Chinese women are deeply troubled by concerns about their physical appearance. Simultaneously, for Chinese women, appearance pressure from Chinese or Asian social media platforms is higher than that from Western media (Jackson et al., 2020).

The body positivity movement is not only popular among young people on Western social media, but its influence on Chinese social media is also significant. Since the end of 2020, discussions of the body positivity movement have primarily focused on visually oriented social media platforms (Cwynar-Horta, 2016; Lang et al., 2023), such as Xiaohongshu and Douyin. However, due to different cross-cultural contexts, the hashtag with #bodypositivity are uncommon on Chinese social media, with #rejectappearanceanxiety being a more prevalent label. Lang’s research on the #rejectappearanceanxiety topic on Chinese social media (Xiaohongshu) found that its connotations were consistent with those advocated by body positivity (Lang et al., 2023). Furthermore, another study by Lang revealed that young Chinese women maintain positive attitudes toward and are influenced by body positivity (Lang and Ye, 2024).

While current body-positive content on social media has generally demonstrated positive effects, its potential negative impacts remain a source of concern and controversy. On the one hand, it may reinforce a focus on appearance (Webb et al., 2017) and potentially contribute to increased overweight and obesity (Cohen et al., 2021). Furthermore, concurrently, concerns exist regarding the privacy and cyberbullying faced by body positivity content creators (bloggers) on social media platforms (Lang and Ye, 2024). On the other hand, the commercialization of body positivity on social media platforms has become a focus of skepticism and concern.

### 2.2 Advertising and body positivity and body image

“Standard paradigm” centered around “perfection, thinness, and the need for constant self-improvement” is being abandoned, replaced by a new advertising paradigm that respects women’s autonomy and emphasizes natural beauty and intrinsic value (Cohan, 2001). Brands are increasingly aware of the negative impact of (Danthinne et al., 2022). Consumers are also more inclined to see media portraying diverse body shapes, sizes, ages, and ethnicities, as well as strategies that emphasize function over appearance to promote positive body image (Paraskeva et al., 2017). As a result, more and more brands are shifting their traditional marketing strategies. A prime example is Dove’s “Real Beauty” campaign, launched in 2004. The campaign aimed to address criticism of unrealistic beauty ideals promoted in advertising by portraying “real” women, thereby alleviating the pressure on women to meet unrealistic appearance standards (Murray, 2013). Selensky ‘s study of 475 women found that those who viewed ads with positive body imagery, such as “Dove Real Beauty,” reported higher self-esteem and perceived the ads as conveying positive, inspiring, and empowering messages (Selensky and Carels, 2021). The success of these ads demonstrates that advertising can be commercially successful without objectifying women or reinforcing stereotypes.

As the body positivity movement gained popularity on social media, businesses joined advocates in championing the cause. However, this led to questions about the commercialization of the movement. On the one hand, there was a focus on social media influencers who had attracted large followings through their advocacy of body positivity. Cwynar-Horta (2016) noted that body positivity advocates on social media were increasingly shifting away from promoting genuine body inclusion in their daily presentations, instead using more body-positive discourse for product marketing and self-presentation (Cwynar-Horta, 2016). Cohen’s content analysis of body-positive Instagram posts revealed that over one-third of “body-positive” content was categorized as promoting commercial products or self-promotion, indicating a shift focus from body positivity ideals toward consumer behavior (Cohen et al., 2019b). This suggests that body-positive social media influencers are driven by profit, gradually deviating from and undermining the positive intentions of the movement. Another concern is corporate exploitation of body positivity for marketing purposes. Businesses capitalize on the social movement of body positivity for commercial gain (Lazuka et al., 2020). However, the radical inclusivity and body-function orientation emphasized in the body positivity movement conflict with the profit-seeking logic of commercial enterprises. The latter’s business model relies precisely on individuals continuously anchoring their self-worth and identity to their outward appearance, driving perpetual consumption (Rodgers et al., 2022). Furthermore, for consumers, the commercial promotion of body positivity can trigger skepticism about the motives behind such campaigns and lead to ethical criticism (Brathwaite and DeAndrea, 2022).

Despite criticism and questioning of the commercialization of body positivity, the positive effects of body-positive advertising cannot be ignored. In terms of advertising effectiveness, compared to traditional “thinness is beauty” advertising, body-positive advertising can promote consumer engagement (Bhattacharjee et al., 2024). Regarding psychological impact, empirical research shows that body-positive advertising on social media can improve body satisfaction (Clayton et al., 2017). When advertising content genuinely reflects the diversity of women and encourages them to accept their uniqueness, it can help boost women’s self-esteem and self-worth. Furthermore, using models that are closer to average female body types rather than thin models can reduce the negative impact of unrealistic body ideals, thereby promoting a healthier body image and higher levels of psychological well-being (Clayton et al., 2017; Lou and Tse, 2021), particularly for female consumers (De Lenne et al., 2023). However, existing research also reveals the complexities and contradictions of body-positive advertising. For example, among women with higher BMIs, using models with non-ideal body shapes in advertisements may alleviate their body anxiety and enhance advertising effectiveness, but it may also provoke negative reactions, weakening the advertising effect (Borau and Bonnefon, 2017). The effectiveness also varies across different brand types (Lou and Tse, 2021). This indicates that body-positive advertising does not produce uniformly positive effects for all groups, with individual differences playing a crucial role.

### 2.3 The current study

In the Western context, existing research has revealed the commercialization of the body positivity movement and its multiple impacts on women’s body image. In contrast, on Chinese social media, brands have increasingly adopted “body positivity “discourse in their marketing efforts, but its impact on young women’s body image remains under-researched. On the one hand, Asian women generally face low body satisfaction, which is related to strong sociocultural pressures and a heightened sensitivity to the judgments of others and external idealized beauty standards. Under the influence of Western culture, “ideal beauty” features such as a high nose bridge, a curvaceous figure, and double eyelids are often unattainable and unrealistic for Asian women. On the other hand, with the rise of feminist social and cultural movements in China, younger generations are increasingly aware of the negative impact of media-constructed “ideal beauty” on individuals’ physical and mental well-being, and are expressing more positive attitudes toward self-acceptance and diversity advocated by the body positivity movement. However, no research has examined the impact of body-positive advertising on Asian women’s body image, confirming whether it has a positive effect on body satisfaction and mood.

Therefore, this study aims to examine the impact of “body-positive “brand marketing on Chinese social media on the body image of young women, focusing on whether it has a positive impact on improving body satisfaction, reducing self-objectification, and enhancing body appreciation. By concentrating on young Chinese women, this research not only extends the applicability of body-positive research to non-Western cultural settings but also helps to illuminate the unique mechanisms through which localized marketing strategies shape women’s body image. In doing so, it offers a new perspective for understanding the complex role of body-positive discourse within processes of commercialization.

## 3 Method

### 3.1 Study design and implementation

This study employed an online Randomized Controlled Trial (RCT) design to systematically examine the immediate psychological impact of Body Positivity (BoPo) advertising on young female users within the Chinese social media context. Given that existing research often relies on correlational data—making it challenging to establish a causal link between advertising content and psychological responses—this design significantly enhances internal validity by manipulating ad types, randomly assigning participants, and strictly controlling both exposure time and stimulus presentation. The study focused on young women aged 18–30, as they constitute both a core demographic of social media users and a group highly sensitive to body image issues. Compared to traditional laboratory experiments, the use of an online situational experiment elevates ecological validity, ensuring participants’ responses more closely mirror real-world social media browsing experiences. Therefore, this design aimed to strike a crucial balance between internal and ecological validity. By combining a pre-test, intervention, and post-test structure, it provides a methodologically appropriate pathway for exploring the immediate psychological responses of young women to diverse body narratives.

### 3.2 Participants and testing procedure

This experiment employed a repeated measures design and utilized G*Power 3 (Faul et al., 2007) for *a priori* power analysis. The results indicated that with a medium effect size (*f* = 0.25) and a significance level (*α* = 0.05), a minimum of 42 participants was required to achieve a statistical power of 80%. Participants were recruited through online social media platforms, resulting in an initial sample of 180 young women who reported using such platforms within the past six months. Eight participants who fell outside the targeted 18–30 age range were excluded, leaving 172 valid participants for the final analysis. Participants represented over 30 cities in China, with the majority (83.72%) aged 18–25. Over 74% reported a Body Mass Index (BMI) between 18.5 and 24.9, 8.72% between 25 and 29.9, 13.95% below 18.5, and 3.33% above 30.

This study primarily employed an online experimental approach to examine the immediate impact of body positivity advertising on young women on social media. The experiment was administered via the Wenjuanxing platform. To ensure participants’ rights to informed consent and adherence to the principle of voluntary participation, they were informed of the confidentiality measures regarding the survey content and the purpose of data collection, and signed an informed consent form before the survey commenced.

Participants were randomly assigned to one of three groups, viewing either ads characterized by body positivity advertising, ads characterized by the ideal beauty (thin-ideal), or appearance-neutral landscape images unrelated to the study.

**Figure 1:**
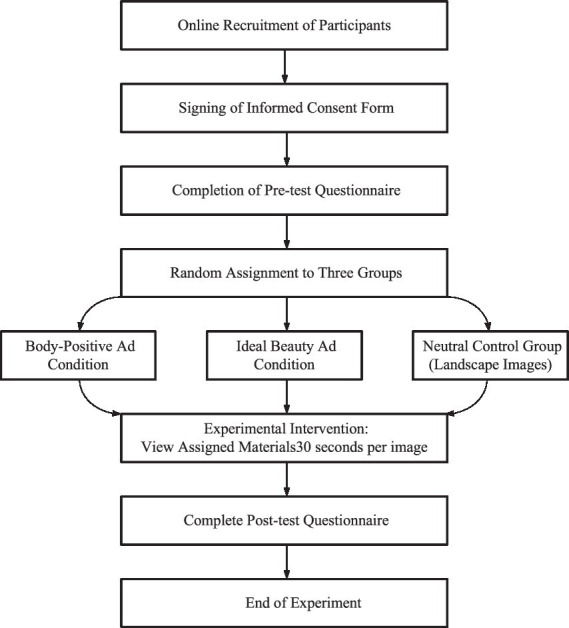
Experimental flowchart.

Before the formal experiment, participants completed a pre-test questionnaire. Subsequently, they entered the experimental phase, viewing the materials sequentially. To ensure experimental validity, the viewing time for each image was fixed at 30 s before the transition to the next stimulus. After viewing, participants immediately completed a post-test questionnaire, thus concluding the entire experimental process. The specific experimental procedure is detailed in Figure 1.

### 3.3 Measures and materials

#### 3.3.1 Materials type

The experimental stimuli and measurements were administered online through the Questionnaire Star platform, with participants automatically assigned to one of the three groups. The stimulus materials consisted of: (1) Advertising posts employing body positivity as a marketing strategy; (2) Advertising posts featuring the ideal beauty (thin-ideal), and (3) A control group with neutral content.

All materials were obtained from public accounts on Xiaohongshu (RED) each condition included 15 posts, presented as images. To facilitate mobile viewing, all images were resized to a 16:9 aspect ratio without altering the original content. Following the procedures used in Cohen’s experimental design, the preparation of materials considered the age and body diversity of the models to ensure ecological validity and generalizability.

In terms of material selection, the study first focused on body-positive advertisements released by brands. Given the absence of explicit body-positive movement hashtags on Chinese social media platforms, this research referred to Lang et al. (2023) and screened hashtags commonly used in body-positive posts on Chinese social media. These included: #身体积极 (BodyPositivity), #拒绝身材焦虑 (RefusingFigureAnxiety), #拒绝容貌焦虑 (RefusingFacialAnxiety), #接纳不完美的自己 (AcceptYourImperfectSelf), among others. Based on these hashtags, Chinese brands were identified, and the top three brands by follower count were selected: @内外 (NEIWAI), @AnActionADay, and @半醒 (Banxing). The main products of these brands encompass underwear (including sports bras), yoga wear, and activewear. Secondly, body-positive advertisements primarily consist of images and textual content. For textual selection, the study chose content emphasizing body functionality—that is, what the body can do. For image selection, materials were controlled based on three aspects: the model’s body type, physical characteristics, and age. Drawing on Lang et al.’s (2023) content analysis method, an analysis of the selected brands revealed the distribution of body types in such advertisements: slightly overweight, overweight, and obese in a ratio of 31:30:4. Regarding whether the images depicted “imperfect” features, 85% of the models displayed at least one characteristic inconsistent with mainstream beauty standards, such as abdominal folds, thick thighs, or wrinkles. In terms of age, the models were predominantly young women.

Based on the above criteria for brand, text, and image selection, a total of 30 body-positive advertisements were chosen. To reduce subjectivity in material selection, an independent researcher specializing in advertising studies was invited to rank the advertisements. Prior to ranking, the researcher received training on the definition of body-positive advertisements to ensure accurate understanding of the concept and enhance the effectiveness of the screening process. Subsequently, the researcher ranked the 30 advertisements, and the top 15 body-positive advertisements were selected as experimental materials. The experimental materials featured 15 models, comprising 7 slightly overweight, 7 overweight, and 1 obese individual; 13 of them exhibited “imperfect” physical characteristics.

The “ideal beauty” posts that experimental materials were searched on Xiaohongshu using keywords such as “clothing” and “advertisement.” Posts containing subjects that met the “ideal beauty” (white, young, and thin) were selected and presented in the same 16:9 format. Similarly, the experimental materials for the neutral group were also selected through the Xiaohongshu public account. They did not contain portraits and mainly involved natural scenery, animals, and plants. The suitability of the experimental materials was verified using the same method for both the “ideal beauty” group and the neutral group. .

**Figure 2:**
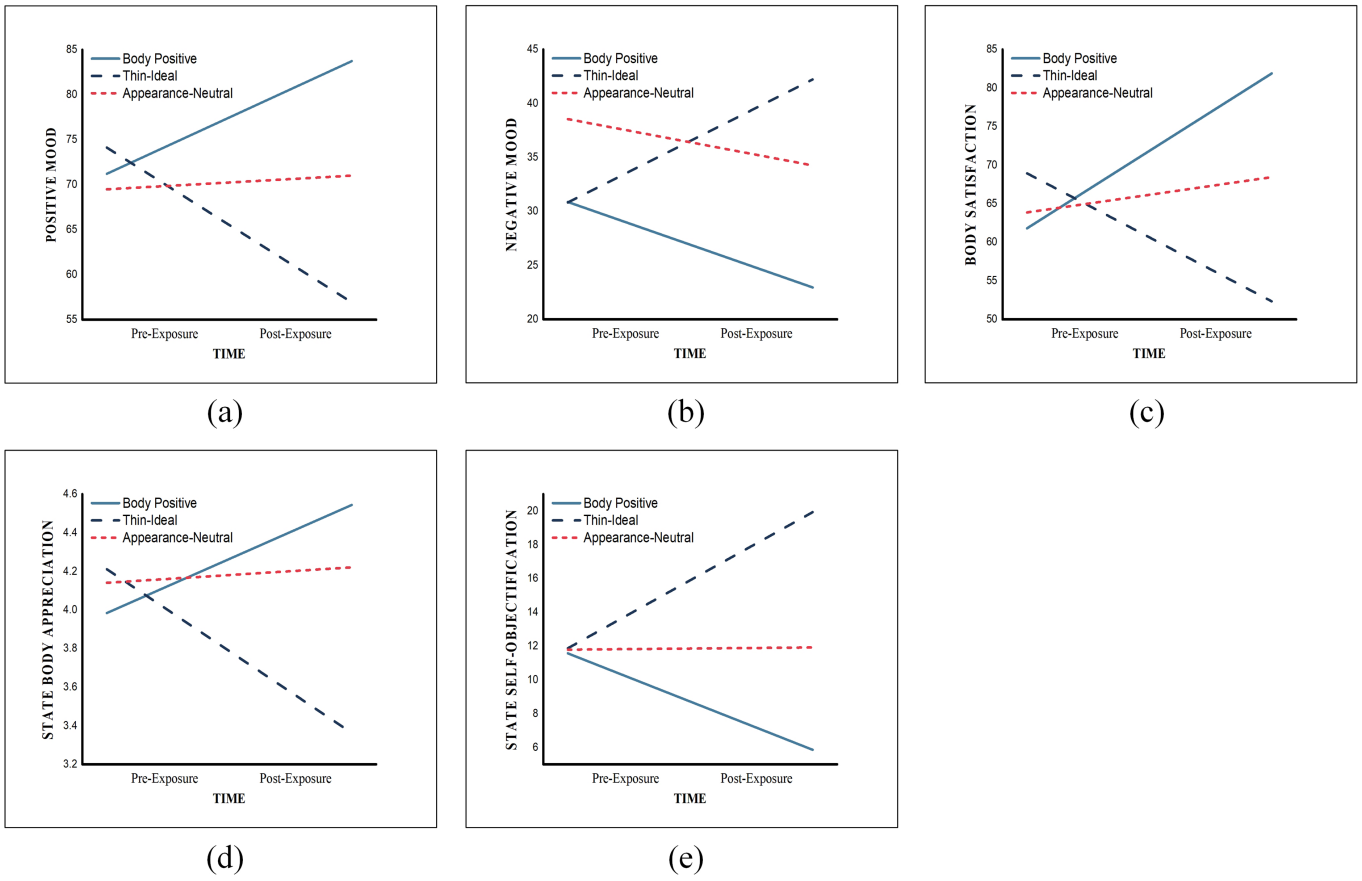
Changes in body image across time for each exposure condition: **(a)** Positive mood; **(b)** Negative mood; **(c)** Body satisfaction; **(d)** Body appreciation; **(e)** Self-objectification.

#### 3.3.2 Measures and materials

##### 3.3.2.1 State mood

The pre-test and post-test were conducted using the Cohen et al. (2019a). The VAS is a reliable and sensitive method for measuring changes in mood and body satisfaction among college women. Participants were asked to rate how they felt “now” by moving a vertical marker to the appropriate point on each horizontal line, with endpoints marked with “not at all” (0) and “very much” (100). Participants were asked to rate a series of mood dimensions: depression, anxiety, confidence, and happiness. Research has found that in low-stress situations, positive and negative emotions are experienced independently and should therefore be measured as separate dimensions (Reich et al., 2003). Therefore, ratings of “happiness” and “confidence” were combined to form a measure of state positive mood, and “depression” and “anxiety “were combined to form a measure of state negative mood (Cohen et al., 2019a). Furthermore, the Visual Analogue Scale (VAS) demonstrated good reliability and sensitivity in measuring changes in female college students’ emotions and physical satisfaction, making it suitable for pre-test/post-test experimental designs. In this study, the pre-test Cronbach’s *α* for positive mood = 0.855, and the post-test Cronbach’s *α* for positive mood = 0.919; the pre-test Cronbach’s *α* for negative mood = 0.753, and the post-test Cronbach’s *α* for negative mood = 0.88.

##### 3.3.2.2 State body satisfaction

Pre-test and post-test were conducted. VAS was used to measure physical satisfaction, which mainly includes the individual’s satisfaction with weight, figure, and overall body in the “current state.” The score ranges from 0 to 100. The higher the score, the higher the satisfaction with the item. The questionnaire score is expressed as the average score of the items (Lang et al., 2023). In this study, the pretest Cronbach’s *α* = 0.885 and the posttest Cronbach’s *α* = 0.924.

##### 3.3.2.3 State body appreciation

The Body Appreciation Scale (BAS) is the most widely used instrument for measuring positive body image, assessing overall positive attitudes toward the body (Halliwell, 2015). The Body Appreciation Scale (BAS-2) was developed by Tylka and Wood-Barcalow and has been translated into 65 countries worldwide. The Mandarin Chinese version of the BAS has also been validated by scholar Ma Jinghua. To avoid translation errors, this paper uses the Mandarin Chinese version of the BAS. In addition, scholars in the same field were invited to review the study beforehand to see if there were any semantic ambiguities due to translation. Because the experiment required immediacy, the State Body Appreciation Scale (SBAS-2) developed by Homan (2016) was combined to measure state body appreciation. The phrase “at this moment “was added before each item, for example, “At this moment, I respect my body.” The scale consists of 10 items, which are scored on a 5-point scale, with 1 = “not at all” and 5 = “very much.” A higher score indicates a higher degree of agreement, and a higher average score indicates a higher degree of body appreciation in the individual. In this study, the pre-test Cronbach’s *α* = 0.932, and the post-test Cronbach’s *α* = 0.967.

##### 3.3.2.4 State self-objectification

The Female Questionnaire of Trait Self-Objectification (FQSO) developed by Wu and Lang (2019) was used for measurement. This questionnaire consists of 17 items, including 10 physical appearance attributes and 7 physical ability attributes. The questionnaire was scored using a 7-point Likert scale, with the physical appearance dimension score minus the physical ability dimension score. This scale has sufficient validity and reliability in assessing self-objectification among Chinese women. In this study, the pre-test Cronbach’s *α* = 0.933, and the post-test Cronbach’s *α* = 0.933.

### 3.4 Data processing

Valid questionnaire data were imported into SPSS 27.0 software for processing and analysis. This study employed repeated measures ANOVA to examine the intervention effects of different advertising types (positive body image advertising, ideal beauty advertising, and a neutral control group) on the body image of young women. Measurement time (pre-test and post-test) was a within-group factor, and advertising group was a between-group factor. In the analysis, demographic variables that may influence body image, such as BMI, were included as covariates in the model to control for potential confounding effects.

With only two levels of within-group variables, the Mochley test for sphericity was not required. The partial *η*^2^ was used as the effect size indicator, representing the degree to which measurement time or advertising type explained the variation in body image. If the interaction effect was significant, a simple effects analysis was conducted to examine the trends in changes between the pre-test and post-test for each advertising group, as well as the differences between groups at the post-test. The Bonferroni method was used to correct for multiple comparisons. The significance level for all statistical tests was set at *α* = 0.05.

## 4 Results

### 4.1 Preliminary analysis

First, one-way ANOVA was used to ensure that there were no initial differences among the participants in the three groups under different experimental conditions: age *F* (2, 169) = 2.858, *p* = 0.06, partial *η*^2^ = 0.033; BMI *F* (2, 169) = 2.238, *p* = 0.11, partial *η*^2^ = 0.026; pre-exposure positive emotion *F* (2, 169) = 0.741, *p* = 0.78, partial *η*^2^ = 0.009; pre-exposure negative emotion *F* (2, 169) = 2.151, *p* = 0.12, partial *η*^2^ = 0.025; pre-exposure body satisfaction *F* (2, 169) = 2.637, *p* = 0.75, partial *η*^2^ = 0.03; pre-exposure body appreciation *F* (2, 169) = 2.121, *p* = 0.123, partial *η*^2^ = 0.024; Self-objectification before exposure *F* (2, 169) = 0.03, *p* = 0.97, partial *η*^2^ < 0.001; Therefore, we can be confident that the groups were homogeneous and comparable in age and BMI before the experiment began. Table 1 shows the mean and standard deviation of each outcome variable under each condition.

**Table 1:** Means (SD) for state positive mood, negative mood, body satisfaction, body appreciation and self-objectification by exposure condition.

Category	Pre-exposure	Post-exposure
Positive mood		
Body positive advertising	71.24 (19.64)	83.71 (14.32)
Ideal beauty advertising	74.02 (17.56)	57.19 (23.76)
Neutral control group	69.57 (22.23)	70.81 (21.95)
Negative mood		
Body positive advertising	30.86 (23.95)	23.14 (24.73)
Ideal beauty advertising	30.86 (21.65)	41.47 (27.36)
Neutral control group	38.52 (23.32)	34.82 (23.22)
Body satisfaction		
Body positive advertising	61.63 (21.11)	81.76 (14.37)
Ideal beauty advertising	69.66 (14.77)	52.87 (23.17)
Neutral control group	63.31 (22.46)	68.04 (20.06)
Body appreciation		
Body positive advertising	3.98 (0.78)	4.54 (0.44)
Ideal beauty advertising	4.24 (0.49)	3.38 (0.89)
Neutral control group	4.10 (0.70)	4.20 (0.70)
Self-objectification		
Body positive advertising	11.56 (7.32)	5.87 (13.02)
Ideal beauty advertising	11.91 (7.68)	19.88 (11.07)
Neutral control group	11.76 (7.69)	11.97 (8.83)

### 4.2 Data analysis

#### 4.2.1 State positivity mood

A repeated measures analysis of variance (ANOVA) was conducted, with advertising type as the between-subjects variable and time (pre-exposure, post-exposure) as the within-subjects variable, while controlling for BMI as a covariate to account for its potential confounding effects. The analysis was performed on the dependent variable, positive mood, to determine whether changes in positive mood over time differ among individuals exposed to different types of advertisements.

The results indicated that, after controlling for BMI, the main effect of measurement time was not significant, *F* (1, 168) = 1.042, *p* = 0.309, partial *η^2^* = 0.006, suggesting that exposure to advertisements itself did not induce a general change in positive mood. In the repeated measures ANCOVA, the interaction effect between time and BMI was not significant, *F* (1, 168) = 1.64, *p* = 0.20, partial *η^2^* = 0.006, indicating that the effect of BMI on positive mood remained stable before and after advertisement exposure. This result satisfied the assumption of “homogeneity of regression slopes” for ANCOVA, thus justifying the inclusion of BMI as a covariate in the model.

The interaction effect between advertisement type and time on positive mood was statistically significant, *F* (2, 168) = 41.628, *p* < 0.001, partial *η^2^* = 0.331. As shown in Figure 2a, simple effects analysis revealed that for participants exposed to body-positive advertisements, their positive mood after advertisement exposure (*M* = 83.773, *SD* = 2.694) was significantly higher than before exposure (*M* = 71.210, *SD* = 2.694), with a mean difference of −12.56, 95% *CI* [−17.21, −7.92], *p* < 0.001. In contrast, for those exposed to ideal beauty (thin-ideal) advertisements, positive mood after exposure (*M* = 56.926, *SD* = 2.72) was significantly lower than before exposure (*M* = 74.128, *SD* = 2.644), with a mean difference of 17.20, 95% *CI* [12.65, 21.76], *p* < 0.001. Comparatively, for participants who viewed appearance-neutral posts, no significant difference in positive mood was observed before and after exposure (*ΔM* = −1.52, 95% *CI* [−6.02, 2.98], *p* = 0.506). All pairwise comparisons were adjusted using the Bonferroni correction to control for multiple comparison errors.

#### 4.2.2 State negative mood

Repeated measures ANOVA was used, with advertising type as the between-group variable and time as the within-group variable, and BMI as a covariate to control for potential confounding effects, to analyze the dependent variable, negative mood, to determine whether the negative mood of people exposed to different types of advertisements changed differently over time.

The results showed that, in the preliminary repeated measures covariance analysis, the interaction between ad type and time on negative mood was statistically significant, *F* (2, 168) = 12.249, *p* < 0.001, partial *η^2^* = 0.127. As shown in Figure 2b, simple effect analysis revealed that for those exposed to positive body image advertisements, the negative mood after exposure (*M* = 30.873, *SD* = 3.109) was significantly lower than before exposure (*M* = 22.955, *SD* = 3.372), with a mean difference of 7.919 and a 95% *CI* of [2.07, 13.767], *p* = 0.008. However, for those exposed to advertisements promoting thinness as beauty, the negative mood after exposure (*M* = 42.217, *SD* = 3.308) was significantly higher than before exposure (*M* = 30.822, *SD* = 3.05), with a mean difference of −11.396 and a 95% *CI* of [−17.134, −5.657], *p* < 0.001. In contrast, no significant difference in negative mood was observed before and after exposure for those viewing appearance-neutral posts (*ΔM* = 4.295, 95% *CI* [−1.376, 9.966], *p* = 0.137). All pairwise comparisons were corrected using the Bonferroni correction to control for multiple comparison error.

It is noteworthy that the interaction effect between time and BMI was significant in the repeated measures covariance analysis, *F* (1, 168) = 4.672, *p* = 0.032, partial *η^2^* = 0.027. To test whether BMI moderates the effect of advertising exposure on body image, a new mixed AMA of 2 (time) × 3 (advertising type) × 2 (BMI) was designed instead of using BMI as a covariate. The results showed that the third-order interaction effect of time × advertising type × BMI was not significant, *F* (6, 160) = 1.8, *p* = 0.102, partial *η^2^* = 0.063. This indicates that, regarding the negative mood of the participants, this pattern of “BMI moderating the time effect” did not vary systematically among the three advertising types. At this point, the participants were simplified into low BMI and high BMI groups according to the WHO BMI reference standard, and the “time main effect” was compared separately. Paired-samples t-tests showed no significant difference in negative mood after viewing the advertisement in the low BMI group (*Mpre* = 33.52, *SD* = 23.55; *Mpost* = 32.81, *SD* = 25.63), *t* (151) = 0.365, *p* = 0.715, *d* = 0.03; while the negative mood in the high BMI group showed an upward trend after viewing the advertisement (*Mpre* = 33.23, *SD* = 20.21; *Mpost* = 37.25, *SD* = 30.05), but did not reach statistical significance *t* (19) = 1.02, *p* = 0.321, *d* = 0.23. The overall interaction was significant while the group effect was not, which may be related to the smaller sample size of the high BMI subgroup and the reduced statistical power. Current data suggest that BMI may be a moderating variable, but its specific effect pattern, especially in the high BMI group, still needs to be confirmed by studies with larger samples.

#### 4.2.3 State body satisfaction

Repeated measures ANOVA was used, with advertising type as the between-group variable and time as the within-group variable. BMI was used as a covariate to control for potential confounding effects. The dependent variable, State body satisfaction, was analyzed to determine whether the changes in State body satisfaction over time differed among people exposed to different types of advertising.

The results showed that, after controlling for BMI, the main effect of measurement time was not significant, *F* (1, 168) = 0.011, *p* = 0.916, partial *η^2^* < 0.001, indicating that advertising exposure itself did not cause a general change in body image. In repeated measures covariance analysis, the interaction effect between time and BMI was not significant, *F* (1, 168) = 0.524, *p* = 0.47, partial *η^2^* = 0.111, indicating that the effect of BMI on body satisfaction remained stable before and after advertising exposure. Importantly, the interaction effect between time and advertising type was significant, *F* (2, 168) = 69.067, *p* < 0.001, partial *η^2^* = 0.451, and the effect size was large, suggesting that the experiment’s impact on body satisfaction depends on the type of advertising viewed.

Due to the significant interaction, we further conducted a simple effects analysis. As shown in Figure 2c, for participants exposed to positive body image advertisements, their body satisfaction after exposure (*M* = 81.891, *SD* = 2.635) was significantly higher than before exposure (*M* = 61.806, *SD* = 2.63), with a mean difference of −20.085, 95% *CI* [−24.493, −15.677], *p* < 0.001; while for participants exposed to advertisements promoting thinness as beauty, their body satisfaction after exposure (*M* = 52.341, *SD* = 2.585) was significantly lower than before exposure (*M* = 68.934, *SD* = 2.581), with a mean difference of 16.593, 95% *CI* [12.268, 20.919], *p* < 0.001. In contrast, those who viewed appearance-neutral posts also showed a significant difference in body satisfaction before and after exposure (*ΔM* = −4.577, 95% *CI* [−8.852, −0.303], *p* = 0.036). All pairwise comparisons were corrected using Bonferroni to control for multiple comparison error.

#### 4.2.4 State body appreciation

Repeated measures ANOVA was used, with advertising type as the between-group variable and time as the within-group variable. BMI was included as a covariate to control for potential confounding effects. The dependent variable, State body appreciation, was analyzed to determine whether the changes in State body appreciation over time differed among individuals exposed to different types of advertising.

The results showed that, after controlling for BMI, the main effect of measurement time was not significant, *F* (1, 168) = 1.418, *p* = 0.235, partial *η^2^* = 0.22, indicating that advertising exposure itself did not cause a general change in Body Appreciation. In the repeated measures ANOVA, the interaction effect between time and BMI was not significant, *F* (1, 168) = 0.679, *p* = 0.411, partial *η^2^* = 0.004), indicating that the impact of BMI on Body Appreciation remained stable before and after advertising exposure. The interaction between ad type and time on body appreciation was statistically significant, *F* (2, 168) = 71.309, *p* < 0.001, partial *η^2^* = 0.459. As shown in Figure 2d, simple effects analysis revealed that for those exposed to positive body image advertising, the body appreciation after exposure (*M* = 4.544, *SD* = 0.095) was significantly higher than before exposure (*M* = 3.984, *SD* = 0.089), with a mean difference of −0.56 and a 95% *CI* of [−0.728, −0.391], *p* < 0.001. However, for those exposed to advertisements promoting thinness as beauty, the body appreciation after exposure (*M* = 3.363, *SD* = 0.093) was significantly lower than before exposure (*M* = 4.211, *SD* = 0.087), with a mean difference of 0.848 and a 95% *CI* of [0.683, 1.014], *p* < 0.001. In contrast, no significant difference in body appreciation was observed before and after exposure for those viewing appearance-neutral posts (*ΔM* = −0.08, 95% *CI* [−0.243, 0.084], *p* = 0.337). All pairwise comparisons were corrected using Bonferroni to control for multiple comparison error.

#### 4.2.5 State self-objectification

Repeated measures ANOVA was used, with advertising type as the between-group variable and time as the within-group variable. BMI was included as a covariate to control for potential confounding effects. The dependent variable, Self-objectification, was analyzed to determine whether the state of self-objectification changed differently over time for individuals exposed to different types of advertising.

The results showed that, after controlling for BMI, the main effect of measurement time was not significant, *F* (1, 168) = 0.069, *p* = 0.792, partial *η*^2^ < 0.001, indicating that advertising exposure itself did not cause a general change in Self-objectification. In the repeated measures ANOVA, the interaction effect between time and BMI was not significant, *F* (1, 168) = 0.354, *p* = 0.553, partial *η*^2^ = 0.002, indicating that the effect of BMI on Self-objectification remained stable before and after advertising exposure. The interaction between ad type and time on self-objectification was statistically significant, *F* (2, 1,168) = 28.125, p < 0.001, partial *η*^2^ = 0.251. As shown in Figure 2e, simple effects analysis revealed that for those exposed to positive body image advertising, the self-objectification score after exposure (*M* = 5.859, *SD* = 1.496) was significantly lower than before exposure (*M* = 11.573, *SD* = 1.024), with a mean difference of 5.714 and a 95% *CI* of [3.12, 8.309], *p* < 0.001. However, for those exposed to advertisements promoting thinness as beauty, the self-objectification score after exposure (*M* = 3.363, *SD* = 0.093) was significantly higher than before exposure (*M* = 19.936, *SD* = 1.468), with a mean difference of −8.062 and a 95% *CI* of [−10.608, −5.517], *p* < 0.001. In contrast, no significant difference in self-objectification was observed before and after exposure for those viewing appearance-neutral posts (*ΔM* = −0.13, 95% *CI* [−2.646, 2.385], *p* = 0.919). All pairwise comparisons were corrected using the Bonferroni correction to control for multiple comparison error.

## 5 Discussion

The main purpose of this study was to examine the impact of body-positive advertisements on social media on young women’s body image, compared to ideal beauty (thin-ideal) advertisements and appearance-neutral posts. This study aims to explore the efforts and contributions of brands in influencing the body image of young women at a psychological level. The findings revealed that brief exposure to body-positive advertising was associated with increased positive mood, body satisfaction, and body appreciation among young Chinese women. In contrast, exposure to thin-ideal advertising produced the opposite effect. Importantly, the study found that brief exposure to body-positive advertising was related to a decrease in self-objectification, which is inconsistent with prior research on body-positive content on social media.

The positive impact of body-positive advertising on young women’s body image aligns with the hypotheses and results of Cohen’s research on body-positive social media posts. First, the results of this study are consistent with the core tenets of Positive Body Image Theory. This theory posits that media content presenting body diversity and emphasizing self-acceptance and authentic bodily experiences can encourage viewers to develop broader concepts of beauty and enhance body image by reinforcing self-care, self-acceptance, and appreciation of body functionality (Tylka and Wood-Barcalow, 2015). Although this study only examined immediate effects and did not explore psychological mechanisms, future research could incorporate variables such as broad conceptualizations of beauty, self-compassion, and self-acceptance to further elucidate the underlying processes. From a psychological perspective, body-positive advertising can effectively improve women’s body image. This finding provides empirical evidence and strategic insights for brands aiming to communicate positive body concepts in their marketing practices. Second, the study shows that body-positive advertisements released by brands do not diminish the positive effects of the body-positive movement due to their commercial nature, addressing scholars’ concerns about the commercialization of body positivity. This study expands the discussion on the commercialization of body positivity: previous research indicated that when body-positive bloggers on social media promote products, consumers easily identify their commercial intentions and attribute the motivation to commercial purposes, considering this practice unethical, thus reducing the effectiveness of body-positive messages (Brathwaite and DeAndrea, 2022). However, in the context of body-positive advertisements published on official brand social media accounts, their overtly commercial nature appears to reduce consumer psychological resistance, allowing the advertisements to exert a more effective positive influence on body image. Furthermore, ideal beauty (thin-ideal) advertising was found to trigger negative affect, increase body dissatisfaction, reduce body appreciation, and reinforce self-objectification, supporting the propositions of the sociocultural model and objectification theory regarding the harmful effects of thin-ideal media. The results confirm that advertising, as a significant medium constructing the thin ideal, has adverse psychological impacts on young women.

Contrary to previous research, this study observed a decrease in self-objectification among women following exposure to body-positive advertisements released by b This finding diverges from the effects typically associated with body-positive content on social media: previous research indicated that even when such content improves mood, body satisfaction, and body appreciation, it may still heighten self-objectification, suggesting that content emphasizing body positivity or the “thin-ideal “can further reinforce focus on appearance (Cohen et al., 2019a; Nelson et al., 2022). However, this study shows that brand-led body-positive advertising did not lead to an increase in self-objectification. Possible reasons include the following: First, this study posits that effective body-positive advertising shifts the informational emphasis away from the “visual attributes” centered on non-idealized models. Unlike advertisements that merely display physical characteristics (e.g., “cellulite” or “abdominal fat”) or non-ideal body types, effective body-positive campaigns typically integrate body functionality (e.g., copy emphasizing bodily capabilities and performance) with product attributes (e.g., highlighting the comfort and support offered by the branded product). This function-product synergy encourages consumers to transition from focusing on “how the body looks” to considering “what the body can do” and “how the product facilitates these functions.” This mechanism aligns with Alleva’s proposition that directing attention to bodily functionality can reduce self-objectification (Alleva et al., 2015). It also supports the positive effects of functionality-focused interventions (Song et al., 2025). Furthermore, these findings extend the discussion initiated by Naidu et al. (2023). While Naidu and colleagues observed that, in the context of health-related products, adopting a functional advertising strategy that frames the “body as a process” can promote health behaviors and purchase intentions, the present research further reveals that such an approach also contributes to reducing self-objectification tendencies. This conclusion resonates directly with the long-standing advocacy of the body-positive movement: shifting public attention from physical appearance to bodily functionality.

Second, the effect of body-positive content on self-objectification may vary across media types. Previous research often examined body-positive content or advertisements posted by social media influencers. Such influencer-generated content, often featuring selfies or images with high degrees of body exposure, inherently adheres to a logic that treats the body as a visual object for display—even when paired with messages of body acceptance. This aligns with findings that “selfie “behavior is perceived as a body image threat by young women (Fox et al., 2021). Additionally, the interactive mechanisms and engagement-driven logic of social media may encourage imitation among audiences, potentially increasing self-objectification. Future research could expand to investigate the effects of body-positive content published across different media platforms. Finally, this study employed a standardized scale to measure self-objectification rather than a declarative self-assessment method, a methodological choice that may have somewhat increased the influence of social desirability bias.

The findings of this study have clear practical implications, providing an effective media-based approach to improving and intervening in women’s body image concerns. Based on data from the Chinese context, this study provides empirical support for the global body positivity movement, demonstrating that brands presenting body diversity in advertising, advocating for a broader definition of beauty, and emphasizing self-acceptance and body function is not only a value proposition but can also genuinely improve the psychological well-being of young women, thus enabling brands to better fulfill their social responsibilities. The research results also respond to the discussion of “body positivity being overly commercialized, “proving that using advertising as a vehicle to convey positive body messages is a feasible and effective strategy that helps alleviate the widespread body image difficulties among young women.

The results of this study should be considered in light of several limitations. First, in terms of experimental design and environment, this study used an online experiment to simulate everyday environments and enhance ecological validity. However, this uncontrolled environment also brings the risk of difficulty in monitoring participants’ responses, and relying solely on self-reports may affect the accuracy of the data. Future research could attempt to replicate this study in a more rigorous laboratory setting to balance internal and external validity. In addition, this study only examined immediate effects, and the long-term effects remain unclear. Future research could combine ecological momentary assessment (EMA) or longitudinal tracking designs to explore the dynamic changes and long-term stability of the effects. The second limitation is in variable control; the study did not include individual trait variables in the control category, such as trait body appreciation, trait self-objectification, trait body satisfaction, social media anxiety related to appearance, or trait self-esteem. These individual differences may act as moderating or confounding variables affecting the experimental results of the impact of body-positive advertising on body image; future research should fully consider these potential influencing factors. The third limitation is in sample representativeness; the sample surveyed young women aged 18–30 in China, neglecting other age groups, men, and other diverse samples. For example, previous studies have shown that men also experience similar body image concerns, particularly influenced by the ideal of muscularity portrayed on social media (Rodgers et al., 2022). This means that exploring samples of men is equally important. Therefore, future research should further expand the gender and age coverage of the sample to improve the generalizability and diversity of the research results. Additionally, as the analysis of state negative mood in Section 4 suggests that BMI may serve as a moderator variable, the current study included only approximately 20 participants in the high-BMI subgroup. Such a small sample size may not adequately represent the heterogeneity of the target population, thereby limiting in-depth inferences regarding the mechanisms and boundary conditions of this moderating effect. Consequently, future research should expand the sample in terms of gender, age, and BMI range to enhance the generalizability and diversity of the findings.

## 6 Conclusion

Advertising, as a primary vehicle for shaping and disseminating norms, discourses, and attitudes about “ideal beauty” (Selensky and Carels, 2021), has long exerted a profoundly negative influence on the body image of young women. This raises an important question: could advertising that conveys body-positive content likewise exert a positive impact? To address this, the present study employed the impact of body-positive advertisements released by brands on the body image of young women through an online experimental survey. The findings reveal that exposure to body-positive advertisements on social media can enhance positive mood, body satisfaction, and body appreciation, and reduce self-objectification levels. The research results not only demonstrate the crucial role of advertising in constructing body image but also provide empirical evidence for alleviating the psychological distress experienced by young women due to the pursuit of unrealistic “ideal beauty” standards. At the same time, this study responds to criticisms of the over-commercialization of the body-positive movement, indicating that brands can convey positive body concepts through advertising, fulfilling their corporate social responsibility while also improving the body image of young women by promoting more positive body concepts.

## Data Availability

The raw data supporting the conclusions of this article will be made available by the authors, without undue reservation.
